# Duct-to-mucosa versus invagination pancreaticojejunostomy after pancreaticoduodenectomy: a meta-analysis

**DOI:** 10.18632/oncotarget.17503

**Published:** 2017-04-28

**Authors:** Shuisheng Zhang, Zhongmin Lan, Jianwei Zhang, Yingtai Chen, Quan Xu, Qinglong Jiang, Yajie Zhao, Chengfeng Wang, Xiaoning Bi, Xiaozhun Huang

**Affiliations:** ^1^ Department of Abdominal Surgical Oncology, National Cancer Center/Cancer Hospital, Chinese Academy of Medical Sciences and Peking Union Medical College, Beijing, 100021, China; ^2^ Department of Obstetrics and Gynecology, Peking Union Medical College Hospital, Chinese Academy of Medical Sciences and Peking Union Medical College, Beijing, 100021, China; ^3^ Department of Abdominal Surgery, Cancer Hospital of Chinese Academy of Medical Sciences, Shenzhen Center, Shenzhen Cancer Hospital, Shenzhen, 518116, China

**Keywords:** duct-to-mucosa, invagination, pancreaticojejunostomy (PJ), pancreaticoduodenectomy (PD), postoperative pancreatic fistula (POPF)

## Abstract

**Objective:**

We aimed to compare the two most commonly used pancreatico-jejunostomy reconstruction techniques—duct-to-mucosa and invagination.

**Methods:**

Databases, including MEDLINE, EMBASE, Cochrane Library, and several clinical trial registration centers were searched. Randomized controlled trials that compared duct-to-mucosa and invagination pancreaticojejunostomy techniques after pancreaticoduodenectomy were included and analyzed.

**Results:**

In total, seven RCTs were included, involving 850 patients. The difference in postoperative pancreatic fistula rate between the duct-to-mucosa and invagination pancreaticojejunostomy was not significant (RR = 1.03, 95% CI = 0.76-1.39, *P* = 0.86). There was no significant difference in clinically relevant postoperative pancreatic fistula between the two groups (RR = 0.78, 95% CI = 0.15-3.96, *P* = 0.77). The overall morbidity, overall mortality, delayed gastric emptying, intra-abdominal collection, reoperation rate, and length of hospital stay between the two groups were not significantly different. Sensitivity analysis showed that the meta-analysis was stable. Further, no significant publication bias was seen.

**Conclusions:**

Duct-to-mucosa and invagination pancreaticojejunostomy techniques after pancreaticoduodenectomy were comparable in terms of postoperative pancreatic fistula incidence and other parameters.

## INTRODUCTION

Pancreaticoduodenectomy (PD) is the most commonly used standard operation for both benign and malignant diseases in the pancreatic head. After the resection, pancreaticojejunostomy (PJ) is regarded the most crucial and problematic technique [1–8]. The postoperative morbidity rate associated with PD remains high, ranging between 20% and 60% [1, 9]. Major complications associated with PD include postoperative pancreatic fistula (POPF), intra-abdominal collection or abscess, and delayed gastric emptying (DGE). Among these, POPF was the most common and problematic complication, with a frequency ranging from 5% to 40% [10]. All these complications can lead to prolonged hospital stays and increased hospital costs [10].

Multiple methods or techniques were explored to reduce POPF incidence after PD. Moreover, numerous PJ modifications were tested to reduce the morbidity after PD [8, 11, 12]. However, few high-quality randomized controlled trials (RCTs) were conducted to assess these modifications. Thus, the best PJ technique remains unknown.

PJ is commonly used as a reconstruction method in multiple pancreatic centers, and duct-to-mucosa and invagination PJs are two classic techniques [1]; several RCTs were conducted to compare these two. However, the number of subjects was relatively small or their results were difficult to interpret because of the different definitions of POPF [9], varying surgical techniques and modifications, and various perioperative treatments used. A meta-analysis conducted by Hua et al. [13] revealed that invagination PJ technique is not superior to duct-to-mucosa PJ technique in terms of POPF incidence but appears to reduce clinically relevant POPF incidence; however, this only included five RCTs, and when the authors analyzed the clinically relevant POPF, only two studies were included. Whether there is a difference in POPF and clinically relevant POPF between the two PJ techniques remains controversial. Recently, more RCTs are now available or ongoing. A relatively high-quality and high-volume RCT was conducted in China [2], which applied the POPF definition proposed by the International Study Group of Pancreatic Surgery (ISGPS), and the result of which was available online. The aim of this meta-analysis was to compare the duct-to-mucosa and invagination PJ techniques after PD.

## RESULTS

### Included studies/literature search

**Figure 1:**
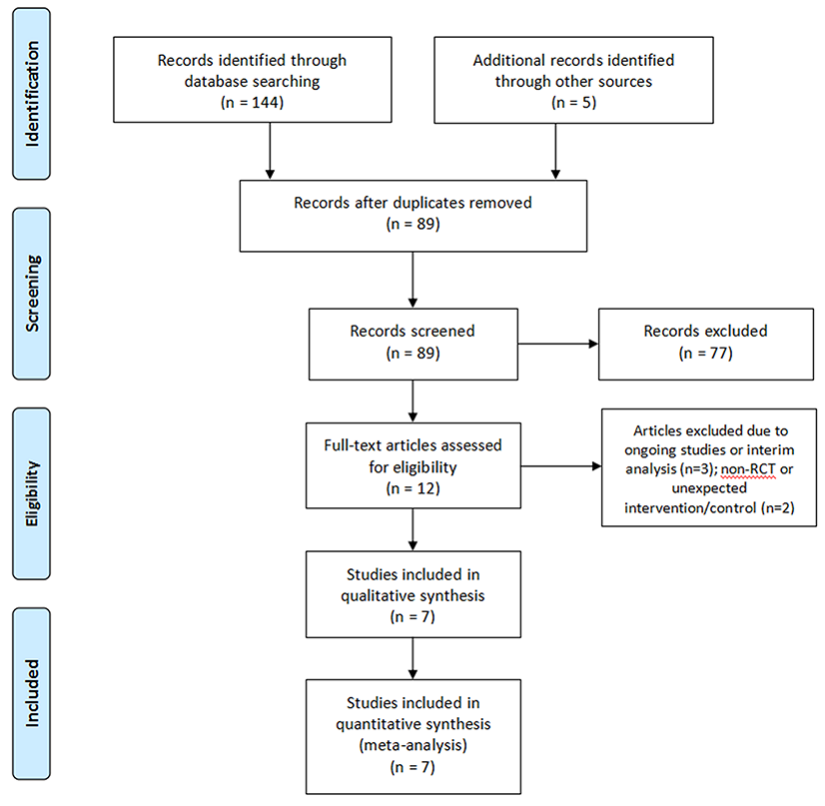
PRISMA flow diagram of the study identification, inclusion, and exclusion A total of 89 non-duplicate records were identified, and finally, seven RCTs were included for the meta-analysis.

A PRISMA flow diagram shown in Figure 1 displays the courses of study identification, inclusion, and exclusion. Initially, online database searching identified 144 records: 51 from MEDLINE, 83 from EMBASE, and 10 from Cochrane Library. The searches in the three platforms, including ClinicalTrials.gov, the Current Controlled Trials Registry, and WHO International Clinical Trials Registry Platform contributed three records. Searches of reference lists for relevant studies, related review articles including meta-analyses, and recent editorials yielded two studies [3, 7] for evaluation. After excluding duplicates, 85 studies remained. After the first title and abstract screening, only 12 records remained. After further screening of the 12 full-text articles, three studies were excluded owing to ongoing studies or interim analyses and two studies because of their non-RCT status or unexpected interventions/controls. Seven RCTs [2–8] were finally included for the quality assessment and meta-analysis.

### Study characteristics

The basic characteristics of the seven RCTs [2–8] are shown in Table 1. The meta-analysis included a total of 850 patients (489 men and 361 women) who underwent PJ after PD from five different countries. Of the 850 patients, 421 (49.5%) underwent duct-to-mucosa PJ and 429 (50.5%) underwent invagination PJ. Table 2 shows the operative details and perioperative managements. Among the 850 patients, 197 patients (46.8%) in the duct-to-mucosa group and 199 patients (47.5%) in the invagination group underwent pylorus-preserving PDs. The mean operative durations were approximately 355.9 min and 347.5 min in the duct-to-mucosa and invagination groups, respectively. Other parameters, such as blood loss, stent placement, pancreatic texture, and somatostatin analogs use varied among the different studies; however, the proportions of these factors in the two groups of each study were comparable.

**Table 1:** Characteristics of the included studies comparing duct-to-mucosa with invagination pancreaticojejunostomy

Reference	Yearof publication	Country	Year ofstudy	Design	No. of pantients (M/F)		Age, mean (range or SD), year		Surgeon
					D-to-M	Inv	D-to-M	Inv	
Bai et al.^2^	2016	China	2012-2015	RCT	64 (38:26)	68 (39:29)	62 (10)	64 (11)	1
Bassi et al.^3^	2003	Italy	1999-2001	RCT	72 (40:32)	72 (46:26)	62 (10)	61 (12)	>1
Berger et al.^4^	2009	USA	2006-2008	RCT	97 (45:42)	100 (54:46)	68 (32-84)	68 (41-90)	8
Chou et al.^5^	1996	China	1984-1996	RCT	47 (23:24)	46 (27:19)	60 (11)	56 (12)	5
El Nakeeb et al.^6^	2015	Egypt	2011-2013	RCT	53 (34:19)	54 (33:21)	54 (12-73)	54 (20-75)	>1
Han et al.^7^	2009	China	2006-2008	RCT	32 (20:12)	32 (24:8)	59 (11)	56 (11)	NA
Langrehr et al.^8^	2005	Germany	1999-2000	RCT	56 (34:22)	57 (32:25)	59 (28-86)	60 (35-79)	>1

RCT: randomized controlled trial; M/F: male/female ratio; D-to-M: duct-to-mucosa; Inv: invagination; SD: standard deviation; NA: data not available.

**Table 2:** Intraoperative and perioperative data of the duct-to-mucosa and invagination groups

Reference		Pylorus preservation	Operative time (min)^a^	Estimated blood loss (ml)^a^	Stents	Pancreatic texture (S/H)	Somatostatin analogs use	Pathology (B/M)
Bai et al.^2^	D-to-M	0	360 (105)	300 (327)	47 used	36/28	2 used	NA
	Inv	0	360 (101)	300 (290)	52 used	44/24	12 used	NA
Bassi et al.^3^	D-to-M	62	379 (63)	NA	Yes	72/0	Yes	18/54
	Inv	65	379 (68)	NA	No	71/0	Yes	23/49
Berger et al.^4^	D-to-M	84	379 (203-698)	500 (100-2000)	Intraoperative temporary^b^	50/47	No	21/76
	Inv	88	347 (204-704)	450 (100-10,000)		51/49	No	34/66
Chou et al.^5^	D-to-M	8	390 (112)	884 (826)	NA	NA	NA	0/47
	Inv	7	326 (78)	1130 (920)	NA	NA	NA	0/46
El Nakeeb et al.^6^	D-to-M	0	330 (180-480)	500 (100-3000)	Intraoperative temporary^b^	25/28	NA	NA
	Inv	0	300 (240-540)	50 (50-2600)		27/27	NA	NA
Han et al.^7^	D-to-M	0	NA	NA	32 used	32/0	NA	NA
	Inv	0	NA	NA	31 used	32/0	NA	NA
Langrehr et al.^8^	D-to-M	43	346 (225-550)	560 (0-2000)	Yes	NA	52 used	18/38
	Inv	39	356 (240-540)	656 (0-2000)	Yes	NA	54 used	14/43

D-to-M: duct-to-mucosa; Inv: invagination; NA: data not available; S/H: soft/hard; B/M: benign/malignant.

^a^Values are expressed as median (range) or mean (standard deviation).

^b^The pancreatic duct stent was inserted during the anastomosis to allow an easy and accurate suture placement, ensure an adequate pancreatic duct exposure, and protect the opposite wall from inadvertently held by needles.

PJ was performed in all participants. The duct-to-mucosa and invagination PJ techniques were performed with or without slight modifications, regardless of the number of layers, end-to-end or end-to-side anastomosis, and types of suture. The definitions of pancreatic fistula (PF) varied among the different trials (Table 3), especially in those conducted before the ISGPS definition was proposed. Four studies [2, 4, 6, 7] applied the ISGPS definition of PF.

**Table 3:** Different definitions of pancreatic fistula among the included studies

Reference	Definition of pancreatic fistula
Bai et al.^2^	ISGPF 2005 definition
Bassi et al.^3^	Output >30 mL/24 hr; rich in amylase content for at least 7 days from post-operative day 4, confirmed by fistulography
Berger et al.^4^	ISGPF 2005 definition
Chou et al.^5^	Drainage of >50 ml/d amylase-rich fluid for >2 weeks
El Nakeeb et al.^6^	ISGPF 2005 definition
Han et al.^7^	ISGPF 2005 definition
Langrehr et al.^8^	Elevated amylase and lipase levels (>1000 U/L) from POD 5 onwards and beyond POD 10, clinical symptoms (pain, fever, etc.)

### Risk of bias and quality of evidence

**Figure 2:**
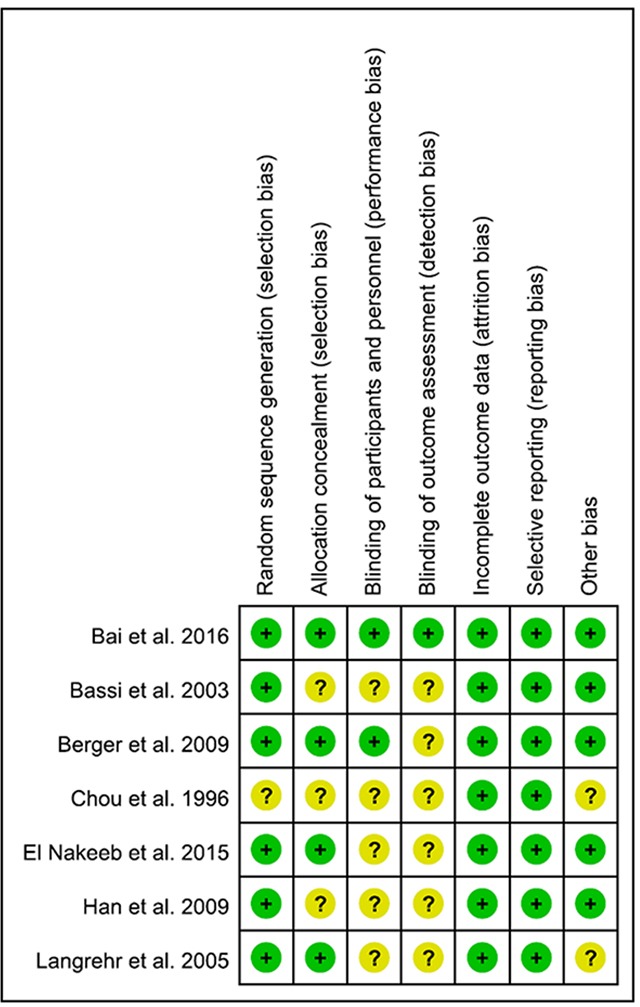
Uality assessment of all included RCTs according to the Cochrane Handbook for Systematic Reviews of Interventions Green circle with a plus sign inside indicates low risk of bias, yellow circle indicates risk, while yellow circle with a question mark inside indicates unable to be assessed risk.

The quality evaluation of all included RCTs is shown in Figure 2. Of the seven included RCTs [2–8], six [2–4, 6–8] were randomized well, and four showed low bias in allocation concealment. Since blinding is quite difficult to apply in surgical studies, further, a division of biostatistics participated in the randomization of two studies [2, 4], showing low risks of participant and personnel bias. Only one study [2] showed a low risk of detection bias. Additionally, attrition bias and reporting bias were good in all studies.

### Primary outcomes/outcomes of interest

#### Postoperative pancreatic fistula

**Figure 3:**
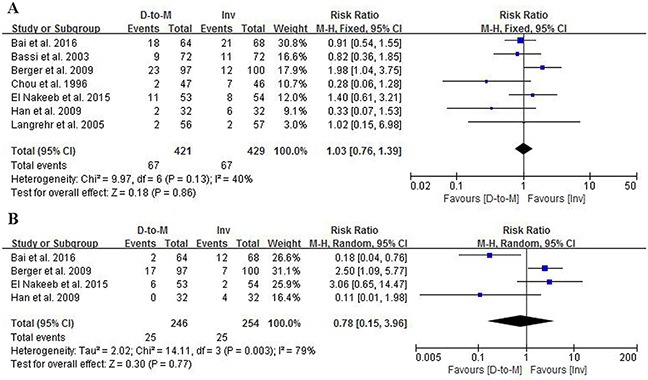
Forest plots comparing the effects of duct-to-mucosa and invagination PJ techniques during PD on POPF **(A)** and clinically relevant POPF **(B)**. D-to-M: duct-to-mucosa; Inv: invagination; M-H: Mantel-Haenszel method.

All the seven RCTs [2–8] reported the POPF rates. The overall POPF rate after PD was 15.9% (67/421) in the duct-to-mucosa group and 15.6% (67/429) in the invagination group. An RR of 1.03 with a 95% CI of 0.76-1.39 was calculated, indicating that no significant POPF difference was present between the two groups (*P* = 0.86). The heterogeneity among the seven RCTs was not statistically significant (Q statistics= 9.97, *P* = 0.13, I^2^ = 40%), and the fixed-effects model was used (Figure 3A).

Four RCTs [2, 4, 6, 7] reported the clinically relevant POPF according to the ISGPS definition, including a total of 500 patients. The overall clinically relevant POPF rates were 10.2% (25/246) and 9.8% (25/254) in the duct-to-mucosa and invagination groups, respectively. There were no significant differences in clinically relevant POPF rates between the two groups (RR = 0.78, 95% CI = 0.15-3.96, *P* = 0.77). There was a significant difference in heterogeneity (Q statistics = 2.02, *P* = 0.77, I^2^ = 79%), and the random-effects model was applied (Figure 3B).

### Secondary outcomes

#### Overall morbidity

**Figure 4:**
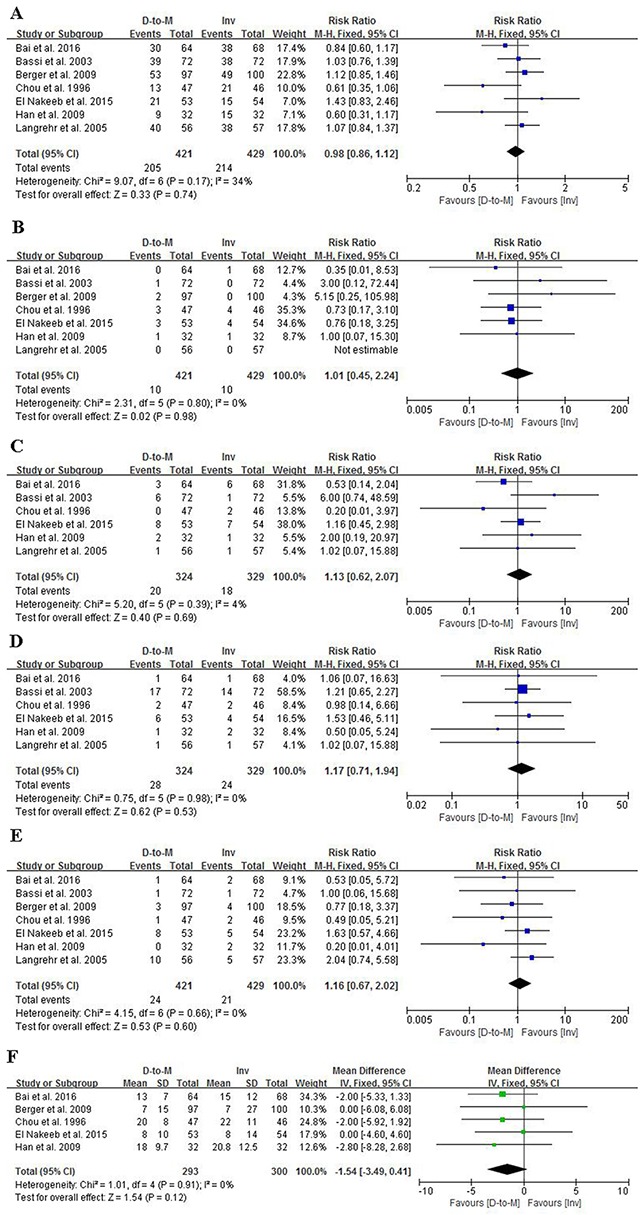
Forest plots comparing the effects of duct-to-mucosa and invagination PJ techniques during PD on overall morbidity **(A)** overall mortality **(B)** DGE **(C)** intra-abdominal collection **(D)** reoperation **(E)** and length of hospital stay **(F)**. D-to-M: duct-to-mucosa; Inv: invagination; M-H: Mantel-Haenszel method; IV: Inverse Variance method; SD: standard deviation.

All included RCTs [2–8] reported the morbidity rates. The overall postoperative morbidity rate among the seven RCTs was 49.3% (419/850), with 48.7% (205/421) in the duct-to-mucosa group and 49.9% (214/429) in the invagination group. There was no significant difference between the two groups (RR = 0/98, 95% CI = 0.86-1.12, *P* = 0.74). No significant heterogeneity was observed in terms of morbidity (Q statistics = 9.07, *P* = 0.17, I^2^ = 34%) (Figure 4A).

#### Overall mortality

The postoperative mortality rates were reported in all the seven RCTs [2–8]. The overall mortality rate was 2.35%, with 2.38% in the duct-to-mucosa group and 2.33% in the invagination group. Data were analyzed using the fixed-effects model, which showed no significant heterogeneity (Q statistics = 2.31, *P* = 0.80, I^2^ = 0%). No significant difference was also observed between the two groups (RR = 1.01, 95% CI = 0.45-2.24, *P* = 0.98) (Figure 4B).

#### Delayed gastric emptying

In most of the RCTs, postoperative DGE was defined as gastric stasis for more than a week. Among the seven RCTs [2–8], only one [4] did not report the postoperative DGE rate. The DGE rate in the duct-to-mucosa group was 6.2% (20/324), and that in the invagination group was 5.5% (18/329). Postoperative DGE rates were analyzed using the fixed-effects model, revealing no significant heterogeneity (Q statistics = 5.20, *P* = 0.39, I^2^ = 4%). An RR of 1.13 and a 95% CI of 0.62-2.07 were observed, showing no difference in postoperative DGE rates between the two groups (Figure 4C).

### Intra-abdominal collection

Six studies [2, 3, 5–8] among the seven RCTs [2–8] reported postoperative intra-abdominal collection. Meta-analysis using the fixed-effects model revealed no significant difference in the postoperative intra-abdominal collection rate between the two groups (RR = 1.17, 95% CI = 0.71-1.94, *P* = 0.53). There was no significant heterogeneity (Q statistics = 0.75, *P* = 0.98, I^2^ = 0%) among the studies (Figure 4D).

#### Reoperation

Reoperations were effective measures for some severe complications, such as severe intra-abdominal bleeding and PF. Reoperation rates were reported in all included RCTs [2–8]. There was no significant difference regarding reoperation rate between the two groups (RR = 1.16, 95% CI = 0.67-2.02, *P* = 0.60), with an overall rate of 5.7% (24/421) in the duct-to-mucosa group and 4.9% (21/429) in the invagination group (Figure 4E). No significant heterogeneity was present (Q statistics = 4.15, *P* = 0.60, I^2^ = 0%).

### Length of hospital stay

Five RCTs [2, 4–7] reported the length of hospital stay with useful data for analysis. No significant difference was found between the duct-to-mucosa and invagination techniques. The MM between the two groups was -1.54 days, with a 95% CI of -3.49 to 0.41 days. No significant heterogeneity was present (Q statistics = 1.01, *P* = 0.91, I^2^ = 0%), and the fixed-effects model was indicated (Figure 4F).

### Subgroup analysis

**Figure 5:**
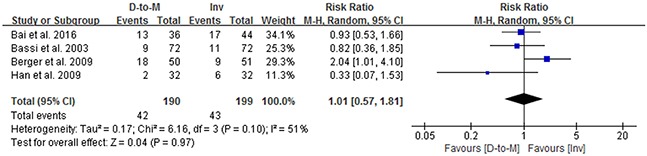
Forest plots comparing the effects of duct-to-mucosa and invagination PJ techniques during PD on POPF in the patients with soft pancreatic remnant texture D-to-M: duct-to-mucosa; Inv: invagination; M-H: Mantel-Haenszel method.

Considering that soft pancreatic texture is a risk factor for POPF, a subgroup analysis for patients with soft pancreas was performed. Four studies [2–4, 7] including 398 patients with soft pancreas were pool analyzed. The POPF rate in the duct-to-mucosa group with soft pancreas was 22.1%, while that in the invagination group was 21.6%, revealing no statistically significant difference. Assessed using the random-effects model and the Mantel-Haenszel method, the RR was 1.01 (95% CI = 0.57-1.81, *P* = 0.97), with a significant heterogeneity among the studies (Q statistics = 0.17, *P* = 0.97) and a corresponding I^2^ statistics of 51% (Figure 5).

### Sensitivity analysis and publication bias

#### Sensitivity analysis

Significant heterogeneities were found among the studies only in terms of clinically relevant POPF and POPF in soft pancreas. Sensitivity analysis was performed by deleting each study or changing the effects model to examine the stability of the results. No significant influence of individual data was found, revealing that our meta-analysis was stable.

#### Publication bias

**Figure 6:**
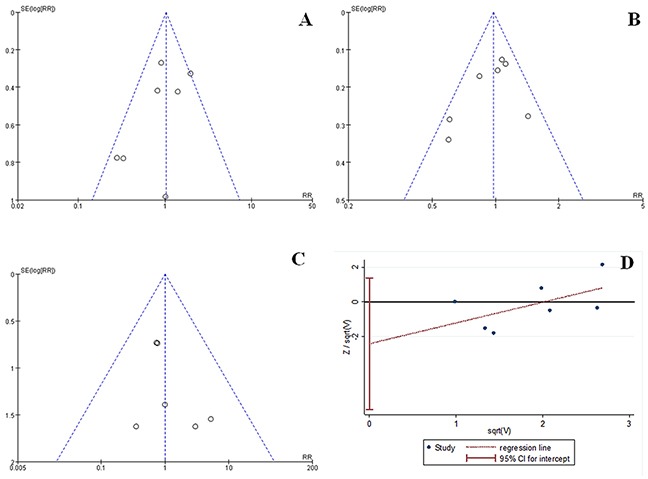
Funnel plots of POPF **(A)** overall morbidity **(B)** and overall mortality **(C)** and Harbord plots of POPF **(D)** revealed no significant publication bias. sqrt: square root calculations; SE(log[RR]): standard error of the natural logarithm of the rate ratio.

The funnel plots based on the POPF and overall morbidity and mortality rates are shown in Figures 6A, 6B, and 6C, respectively. The funnel plot shapes were symmetrical, indicating no obvious reporting bias. The Harbord plots based on the POPF are shown in Figure 6D. No obvious reporting bias was found using the Harbord's modified test (*P* = 0.164).

## DISCUSSION

The pancreaticoenteric anastomosis is often regarded as the crucial step of PD, and many efforts have been made to reduce the associated morbidity and mortality. PJ remains the preferred technique in many multiple high-volume pancreatic centers to reestablish the gastrointestinal and pancreatic continuity after PD. Duct-to-mucosa and invagination PJs are the two most commonly used techniques. However, the optimal technique between these two is debatable in terms of reducing complications, especially POPF.

In this meta-analysis, seven RCTs [2–8] were included. The meta-analysis revealed no significant difference in POPF and clinically relevant POPF rates between the duct-to-mucosa and invagination PJ techniques. For other secondary outcomes of interest, including overall morbidity and mortality, DGE, intra-abdominal collection, reoperation, and length of hospital stay, no significant differences were found between the two techniques. All details of the data are shown in Table 4.

**Table 4:** Summary of the results comparing duct-to-mucosa versus invagination PJ after PD

	No. of studies	No. of patients	Rate ratio	*P* value	*P* forheterogeneity	I^2^(%)
**Primary outcomes**						
Pancreatic fistula	7	850	1.03	0.86	0.13	40
Clinically relevant POPF	4	500	0.78	0.77	0.003*	79
**Secondary outcomes**						
Delayed gastric emptying	6	653	1.13	0.69	0.39	4
Intra-abdominal collection	6	653	1.17	0.53	0.98	0
Overall morbidity	7	850	0.98	0.74	0.17	34
Overall mortality	7	850	1.01	0.98	0.80	0
Reoperation	7	850	1.16	0.60	0.66	0
Length of stay	5	593	MD:-1.63	0.11	0.93	0

* Statistically significant

In duct-to-mucosa PJ, the suture between the duct and mucosa is beneficial for healing. Moreover, the pancreatic remnant is protected by the jejunal serosa. However, the space between the jejunal wall and pancreatic stump may offer a potential place for pancreatic juice retentions from accessory or multiple tiny pancreatic ducts [10]. Additionally, the duct-to-mucosa technique is quite difficult for some pancreas without an enlarged pancreatic duct. On the contrary, the invagination PJ technique is much easier to perform as there is no potential space for pancreatic juice retention, and all the pancreatic juices flow into the bowel [6]. However, this technique may cause pancreatic stump ischemia.

POPF is the primary outcome of interest to assess the two techniques. In our meta-analysis, the different effects of the duct-to-mucosa and invagination techniques on POPF rate were not significant. Among the seven trials, only Berger et al. [4] reported a reduced risk of PF with invagination PJ technique, while others [2, 3, 5–8] did not. In 2005, the POPF definition was proposed by the ISGPS [9]. In this system, grade A PF was defined as PF without clinical impact that required little management and grades B and C PF as those that required more positive interventions, indicating clinically relevant POPFs [4]. However, we failed to find a significant difference in clinically relevant POPF between the two techniques in this meta-analysis (10.2% versus 9.8%, *P* = 0.77). Among the four RCTs, two studies had opposite conclusions. Duct-to-mucosa technique was preferred by Bai et al. [2] and invagination technique by Berger et al. [4] This might be because different surgeons may prefer some special techniques, and their experience may have affected the complication occurrences. Another study conducted in China [7] showed the same tendency.

Recently, the Fistula Risk Score, a grading system based on four parameters (soft pancreatic texture, small pancreatic duct size, pathology, and blood loss) has been proposed [14, 15]. Some retrospective studies [16, 17] showed a better performance in POPF for the duct-to-mucosa technique in patients with hard pancreas, while invagination technique had advantages in patients with soft pancreas. However, in our subgroup analysis, the advantages of the invagination technique in the patients with soft pancreas were not observed. Significant heterogeneity was shown among the studies, which may be caused by the different classifications of pancreatic texture. Moreover, none of the included studies evaluated the duct size, pathology, or blood loss for POPF, which may increase the POPF rate itself. Further RCTs considering all the four factors are needed.

The present meta-analysis showed an overall morbidity rate of 49.3% and mortality rate of 2.35%, which are consistent with those of other studies [18, 19]. The overall morbidity rate varied in different studies [2–8], ranging from 33.6% to 69.0%, possibly because of the diverse definitions of complications, surgical techniques, and other factors. The overall mortality rate also differed among studies [2–8], ranging from 0% to 7.5%. Our analysis did not find any significant difference between duct-to-mucosa and invagination PJ in overall morbidity and mortality rates.

DGE could occur in all abdominal surgeries involving the stomach. This complication is not usually life-threatening; however, it can cause abdominal discomforts, prolonged hospital stays, and higher expenses. Though the definition and grading system of DGE have been proposed by the ISGPS [9], which reflect the clinical features and courses of DGE properly, only one RCT [2] applied the definition clearly when assessing their secondary outcomes. The DGE rate varied among RCTs [2, 3, 5–8], ranging from 1.8% to 14%. In our meta-analysis, the DGE rates between the duct-to-mucosa and invagination groups were similar. On the other hand, intra-abdominal collection could occur in any abdominal surgery, which differs depending on the nature of collection. The incidence of this complication varied from 1.5% to 21.5% [2, 3, 5–8]. Our analysis revealed no significant difference in the postoperative intra-abdominal collection rate between the duct-to-mucosa and invagination groups.

Reoperation rate is usually correlated with mortality and is between 3.1% and 13.3% [2–8]. Reoperation [2–8] is needed when severe complications occur, including bleeding, bile leakage, intra-abdominal collection or abscess, and local peritonitis; many of these complications were not directly associated with POPF. Therefore, reoperation is not a specific outcome for PJ techniques. Furthermore, lack of consensus on indications for reoperation leads to clinical heterogeneities. The decision for reoperation is often made by a senior surgeon by experience. Although the I^2^ test of the RCTs showed no heterogeneity (I^2^ = 0), some clinical heterogeneities were found.

Length of hospital stay also varied among studies [2, 4–7]. It is a secondary outcome related to all kinds of complications, cost, and allocation of medical resources. In our meta-analysis, the duct-to-mucosa technique seemed to shorten hospital stay; however, no significant difference was found. Further, as in reoperation, the length of hospital stay could be affected by the availability and efficiency of medical resources, patient transfer systems, and doctor's habits.

Most clinical studies [2–4, 6–8] were conducted with additional treatments to a certain extent other than the investigated treatments, including the use of internal or external stents, sealants, octreotides, and analogs. Some of these treatments may affect the POPF rate, further introducing clinical heterogeneities. External stents, which have been proven to be able to reduce POPF rate, may affect the true effects of the two PJ techniques on the POPF rate. Therefore, we should pay more attention to the interpretation of the meta-analysis.

The present meta-analysis has some strengths. Firstly, this meta-analysis includes seven RCT [2–8] conducted in different centers in different countries, including 850 subjects, which provides a relatively high-level of evidence. Secondly, this meta-analysis is stable as proven by the sensitivity analysis. Thirdly, no significant publication bias was detected. However, several limitations in the present meta-analysis could not be ignored. Firstly, the duct-to-mucosa and invagination techniques, with or without modifications differed to a certain extent. Secondly, many supplemental techniques, such as external stents and sealants and perioperative managements may affect the outcomes, introducing clinical heterogeneities. Thirdly, when we analyzed the effects of PJ techniques on POPF in the patients with soft pancreatic texture and those on clinically relevant POPF, the number of the included studies and subjects was relatively small, and a significant heterogeneity exists. Further RCTs considering the abovementioned limitations are required before stronger evidence-based recommendations could be made.

## CONCLUSION

In conclusion, duct-to-mucosa and invagination PJ techniques after PD were comparable in terms of POPF and clinically relevant POPF. Subgroup analysis of the effects of the PJ techniques on POPF in soft pancreas revealed no significant difference. No significant difference was also found between the two techniques in multiple secondary outcomes, including overall morbidity and mortality, DGE, intra-abdominal collection, reoperation, and length of hospital stay. Further well-designed, large-volume, multi-center RCTs that would apply more standardized outcome definitions and consider more risk factors comparing the two techniques are still required.

## MATERIALS AND METHODS

### Search strategy and study selection

This meta-analysis was conducted to compare the duct-to-mucosa and invagination PJ techniques following PD and reported according to the Preferred Reporting Items for Systematic Reviews and Meta-Analyses (PRISMA) statement [20].

Several databases, including MEDLINE, EMBASE, and Cochrane Library were searched to identify studies published between November 1945 (the first time when duct-to-mucosa PJ was mentioned) and September 2016. The following words were searched as “text words”: (1) terms suggestive of “duct-to-mucosa” (i.e., “duct-to-mucosa,” “duct to mucosa,” or “cattell”) and (2) “invagination” (i.e., “invagination,” “invaginated,” “invaginating,” “Dunking,” “Duct-invagination,” “Duct-invaginated,” “Duct-invagina- ting,” or “mattress”). The full searching strategy is available as an appendix or from the corresponding author. Several registry platforms were also searched, including ClinicalTrials.gov, the Current Controlled Trials Registry, and WHO International Clinical Trials Registry Platform. The following major academic conferences were also searched: American Society of Clinical Oncology (ASCO), European Society for Medical Oncology (ESMO), and European Society of Surgical Oncology (ESSO). Reference lists of relevant studies, related review articles including meta-analyses, and recent editorials were manually searched for additional studies. For the same abstracts without detailed data and other studies with unpublished data, we contacted the corresponding authors to obtain useful data.

### Inclusion and exclusion criteria

Two authors (Shuisheng Zhang and Zhongmin Lan) independently screened all the records. Titles and abstracts were searched for the first time for potential eligible RCTs that compared the two PJ techniques, and the full texts were searched for the final decision. When any discrepancies arose, a senior surgeon (Chengfeng Wang) served as an arbitrator.

For the inclusion, the studies had to fulfil the following criteria: RCTs comparing the two techniques, published in a peer-reviewed journal, and with outcomes including POPF. Ongoing RCTs or abstracts without useful data, retrospective studies, non- or pseudo-randomized clinical trials, case reports or series, editorials or expert opinions, letters, and reviews were excluded. Those with locally unresectable diseases, metastatic diseases, total pancreatectomy, duodenum-preserving pancreatic head resection or any operation other than PD, other types of anastomosis, or even without pancreaticojejunal anastomosis were excluded.

### Participants and interventions

The participants included for the analysis were those who underwent PJ following PD for any indications. Comparison was made between the duct-to-mucosa and invagination PJ techniques. Invagination procedures with identical modifications, such as end-to-end or end-to-side PJ and different numbers of suture layers were all included after confirmation from the authors.

### Outcomes of interest

The primary outcome was POPF. For studies [2, 4, 6, 7] that applied ISGPS definition [9], which was proposed in 2005, data about clinically relevant POPF (grades B and C) were also extracted and assessed. Secondary outcome measures included overall morbidity and mortality, DGE, intra-abdominal collection, reoperation, and length of hospital stay.

### Data collection

The data were extracted by two reviewers (Qinglong Jiang and Zhongmin Lan) independently. All extracted data were cross-checked, and any inconsistency was discussed and solved with a senior reviewer (Quan Xu) until consensus was reached. Data expressed as mean and standard deviations (SDs) were needed; if such data were not available, we calculated the median and range values [21].

### Evaluation of quality and assessment of risk of bias

The quality of the included RCTs was evaluated by two authors (Shuisheng Zhang and Yajie Zhao) according to the Cochrane Handbook for Systematic Reviews of Interventions [22]. The quality was classified as low, high, or unclear risks. A third reviewer (Xiaoning Bi) joined in when inconsistencies occurred until a final decision was made through discussions.

### Statistical analysis/analysis

Data were analyzed using the Review Manager 5.3 (The Cochrane Collaboration, The Nordic Cochrane Center, Copenhagen, Denmark) and StataSE 12.0 software (StataCorp, College Station, Texas). The Cochrane Q statistics and I^2^ statistics were used to evaluate the heterogeneity among the included RCTs. *P* < 0.1 (Q statistics) and I^2^ > 50% were considered as significant heterogeneities [23]. The fixed-effects model was applied to synthesize the data of the different trials when there were no significant heterogeneities; otherwise, the random-effects model was used [24]. Hospital stays were presented as mean differences (MDs) with 95% confidence interval (CIs). Other results were expressed as rate ratios (RRs) with 95% CIs. Subgroup analysis on POPF was conducted among these RCTs with information on pancreatic texture. The publication bias of the included studies was assessed using the funnel plots based on the primary outcome POPF and several secondary outcomes. Sensitivity analysis was done using the Harbord plots to evaluate the stability of the meta-analysis.

## Abbreviations

ASCO: American Society of Clinical Oncology
Cis: confidence intervals
ESSO: European Society of Surgical Oncology
ESMO: European Society for Medical Oncology
DGE: delayed gasatric emptying
ISGPS: International Study Group of Pancreatic Surgery; MDs: mean differences
PD: pancreaticoduodenectomy
PJ: pancreaticojejunostomy
POPF: postoperative pancreatic fistula
PRISMA: Preferred Reporting Items for Systematic Reviews and Meta-Analyses
RCTs: Randomized controlled trials; risk ratios (RRs); SDs: standard deviations.
